# Visible Light-Mediated Inactivation of H1N1 Virus UsingPolymer-Based Heterojunction Photocatalyst

**DOI:** 10.3390/polym15112536

**Published:** 2023-05-31

**Authors:** Stefania Porcu, Stefania Maloccu, Angela Corona, Moulika Hazra, Tullia Carla David, Daniele Chiriu, Carlo Maria Carbonaro, Enzo Tramontano, Pier Carlo Ricci

**Affiliations:** 1 Department of Physics, University of Cagliari, 09042 Cagliari, Italy; moulika.hazra@unica.it (M.H.); davidtulliacarla@gmail.com (T.C.D.); daniele.chiriu@dsf.unica.it (D.C.); cm.carbonaro@dsf.unica.it (C.M.C.); 2 Department of Life and Environmental Sciences, University of Cagliari, 09124 Cagliari, Italy; stefaniamaloccu@icloud.com (S.M.); angela.corona@unica.it (A.C.); tramon@unica.it (E.T.)

**Keywords:** photocatalytic virucidal activity, environmental disinfection, carbon nitride, hybrid materials, visible light, photocatalysis, environmental remediation

## Abstract

It is well known that viruses cannot replicate on their own but only inside the cells of target tissues in the organism, resulting in the destruction of the cells or, in some cases, their transformation into cancer cells. While viruses have relatively low resistance in the environment, their ability to survive longer is based on environmental conditions and the type of substrate on which they are deposited. Recently, the potential for safe and efficient viral inactivation by photocatalysis has garnered increasing attention. In this study, the Phenyl carbon nitride/TiO_2_ heterojunction system, a hybrid organic–inorganic photocatalyst, was utilized to investigate its effectiveness in degrading the flu virus (H1N1). The system was activated by a white-LED lamp, and the process was tested on MDCK cells infected with the flu virus. The results of the study demonstrate the hybrid photocatalyst’s ability to cause the virus to degrade, highlighting its effectiveness for safe and efficient viral inactivation in the visible light range. Additionally, the study underscores the advantages of using this hybrid photocatalyst over traditional inorganic photocatalysts, which typically only work in the ultraviolet range.

## 1. Introduction

Harmful microorganisms, such as viruses, bacteria, and protozoa, are ubiquitous in our environment and can be found in various settings, such as water sources, soil, and even in the air we breathe. While some of these microorganisms are harmless, others can cause serious health issues if they enter our body and multiply, leading to the development of infectious diseases [1].

Microorganisms that are contracted via food, water, and air, enter the body through different methods of infection, leading to over 15 million deaths annually worldwide. In recent years, the issue of harmful microorganisms in the environment has become increasingly pressing due to various factors such as climate change, urbanization, and the globalization of travel and trade. Climate change, for example, has led to the emergence and spread of new pathogens that were previously confined to specific regions or environments. Urbanization and globalization have increased the density of human populations and facilitated the movement of people and goods, creating conditions that are favorable for the transmission of infectious diseases [2].

To address this issue, it is essential to implement effective measures to control and prevent the spread of harmful microorganisms in the environment. This can include measures such as improving sanitation and hygiene practices, treating and disinfecting water sources, and implementing vaccination programs. Additionally, the development of new technologies and strategies for detecting and controlling harmful microorganisms can also play a crucial role in addressing this issue [3,4]. Furthermore, for the disinfection of waterborne viruses, multiples techniques have been applied to UV disinfection, membrane filtration, and chemical disinfection, but all of them suffer disadvantages (high cost, energy consuming, and health risk) [5,6,7,8].

Oxidizing processes, such as ozonation, photoelectronic oxidation photocatalysis, etc., have been deeply studied as promising strategies to treat virus contamination [9] and, among all the emerging technologies, photocatalysis has been widely studied for its ability to degrade various pollutants and for its potential in medical, environmental applications [10].

In particular, the photocatalytic inactivation of viruses has been the subject of intense research due to the increasing global threat of viral outbreaks [11,12,13,14,15].

This process involves the use of a photocatalyst, which is activated by radiation to generate reactive oxygen species (ROS). ROSs have strong oxidizing power and can react with various biomolecules, including lipids and nucleic acids. The lipid envelope of the virus is essential for its survival and the infection of host cells. When the virus comes into contact with ROS, the ROS can cause damage to the lipid envelope, leading to the loss of structural integrity and function. Additionally, ROS can react with the nucleic acid of the virus, causing damage to the DNA or RNA and rendering it unable to replicate or infect host cells. One of the advantages of photocatalytic inactivation is that it is a physical process which does not require the addition of chemicals, making it a potentially safer and more environmentally friendly approach to virus control. Additionally, the photocatalytic mechanism has been shown to be effective against a wide range of viruses, including those that are highly infectious and resistant to traditional disinfection methods [16,17].

Photocatalytic efficiency depends on the recombination of the photogenerated charges (the slower the recombination, the better) and on the capability to use solar light for the process. Most semiconductor photocatalysts studied until now (g-C_3_N_4_, Ag_3_PO_4_, TiO_2_, ZnO) do not satisfy all these requirements [18,19], and different approaches have been made to modify the structures for improving the photocatalytic efficiency, such as doping and surface modification coupling with other semiconductors [20,21].

Among the others’ two-dimensional conjugated polymers have emerged as a promising class of materials for photocatalysis due to their eco-friendliness, abundance of constituent elements, ease of synthesis, and chemical stability. In particular, an organic–inorganic hybrid system that combines TiO_2_ with graphitic carbon nitride (g-C_3_N_4_) has attracted interest because it allows for the synergistic use of TiO_2_’s photocatalytic properties with g-C_3_N_4_’s lower band gap energy (2.7 eV) and thermal and chemical stability [22,23]. The delocalized conjugated structure of g-C_3_N_4_ contributes to a slow charge recombination rate and rapid photoinduced charge separation. By forming a charge-transfer complex at the interface between the organic donor (g-C_3_N_4_) and inorganic acceptor (TiO_2_), the g-C_3_N_4_/TiO_2_ heterojunction system can decrease the recombination rate of photogenerated electron-hole pairs, as well as increase the photocatalytic activity of TiO_2_ under visible light for wavelengths as low as 450 nm [24,25].

The band scheme of the band structure of TiO_2_/g-C_3_N_4_ can be seen as an efficient Z-scheme, in which an internal electric field is directed from g-C_3_N_4_ to TiO_2_ due to their different work functions. This allows for the efficient separation and transfer of photogenerated charge carriers, leading to enhanced photocatalytic performances. Overall, the use of g-C_3_N_4_/TiO_2_ hybrid systems presents a promising avenue for designing efficient and stable photocatalytic materials with potential applications in environmental remediation, energy conversion, and other areas. The only concern is the use of UV light to start the reaction and, therefore, the low efficiency of solar, natural light and most interior lights. UV light has been widely used for disinfection due to its germicidal properties, but UV radiation, particularly in the UVC range, can be harmful to living organisms, including humans. Prolonged exposure to UVC light can cause damage to the skin and eyes. In this study, we aim to highlight the promising use of Phenyl carbon nitride/TiO_2_ (hereafter, PHCN refers to phenyl carbon nitride) in indoor environments, where UV lighting is not allowed, and solar illumination may not always be available. We emphasize the applicability of PhCN/TiO_2_ in indoor settings and the advantages of visible light sources such as LEDs. By utilizing visible light and focusing on the use of LEDs typically employed for indoor lighting, we can harness their energy-efficient and controllable properties to activate the PhCN/TiO_2_ photocatalysis effectively. By emphasizing the applicability of PhCN/TiO_2_ in indoor settings and the advantages of visible light sources such as LEDs, we can pave the way for the practical and safe implementation of PhCN/TiO_2_ photocatalyst for various indoor applications [26]. The organic component, benzene carbonitrile, exhibits a broad absorption band in the visible region and a bandgap of 2.0 eV, which makes it an excellent candidate for extending the spectral range of titanium dioxide in coupled systems. Actually, the efficiency of phenyl carbon nitride as a standalone photocatalytic material is poor due to the high recombination rate of photogenerated electron–hole pairs. This interaction between the two structures is crucial for facilitating charge transfer and separation to allow the activation of the photocatalytic process under visible light irradiation. In the photocatalytic processes, the PhCN acts as a sensitizer, and, once visible radiation is absorbed, charges are first transferred from the valence band to the conduction band of the organic component and subsequently to the conduction band of the titanium dioxide, which activates the photocatalytic mechanism. Due to its characteristics, it appears as a promising virucide agent and in this work, we focus the discussion on the photocatalytic inactivation of H1N1 under visible light irradiation.

## 2. Materials and Methods

### 2.1. Materials

Ph-Triazine (99%), titanium (IV) chloride (99%), were purchased from Sigma Aldrich, St. Louis, MO, USA. Absolute ethanol (94–96%) was sourced from Alfa Aesar, Haverhill, MA, USA. All chemicals were used as received without further purification.

#### Cells and Viruses

Madin-Darby canine kidney cells (MDCK-2 ATCC^®^ CRL-2936™, Merck, Darmstadt, Germany) were maintained in DMEM (gibco) supplemented with 10% heat-inactivated Fetal Bovine Serum (FBS HI) (gibco) and 1% Kanamycin (KAN) sulfate. Influenza virus strain A/Puerto Rico/8/34 (H1N1) was propagated in MDCK-2 Cells. In total, 4 × 10^6^ cells were seeded in a 75 cm^2^ flask in 20 mL complete medium and seed at 37 °C, 5% CO_2_ for 24 h. Then, the supernatant was discarded, and cells were rinsed with 10 mL of PBS and infected for 1 h with 1 mL of viral dilution (m.o.i. 0.01) in DMEM + 1% KAN. After 1 h, 10 mL of maintaining media (DMEM+ 1% KAN + 1% FBS HI) was added and infection was incubated for 72 h. Then, cells were cryolised with 3 cycles of 30 min at −80 °C/30 min 37 °C and pelleted to discard cellular debris 20 min at 3000 G. Supernatant was stored at −80 °C. Viral stock was titrated by plaque assay.

### 2.2. Synthesis of the Photocatalyst

#### 2.2.1. Synthesis of PhCN

PhCN was prepared by placing 1 g of 6-phenyl-1,3,5-triazine-2,4-diamine powder (Ph-Triazine) in a quartz tube accommodated in a tubular furnace and heated to 400 °C for 2 h [27].

#### 2.2.2. Synthesis of PhCN/TiO_2_

A total of 100 mg of prepared PhCN was stirred at room temperature for 30 min in 20 mL of ethanol, and then 0.5 mL of TiCl_4_ was added, and the solution was stirred at room temperature for 2 h. The solution was transferred into a Teflon autoclave and heated at 180 °C for 8h. The product was filtered and washed multiple times with DI water and absolute ethanol and dried at 60 °C for 12h [26].

### 2.3. Characterization Techniques of the Photocatalyst

X-ray diffraction patterns were collected at room temperature by using a Rigaku Miniflex II diffractometer (Rigaku, Tokyo, Japan) with θ–2θ Bragg-Brentano geometry with Cu Kα (λ = 1.5418 Å), radiation at room temperature. The powder patterns were collected in the 2θ range of 10° to 90°.

Absorption measurements were obtained by diffuse reflectance spectroscopy utilizing a UV–Vis–NIR Agilent Technologies Cary 5000 Spectrometer (Santa Clara, CA, USA). Measurements were performed by using a PbS solid-state photodetector. The reflection configuration measures the diffuse reflection of the sample with respect to a KBr reference that is considered to have 100% reflectivity. The Kubelka-Munck equation was applied to extract the absorption features.

#### Cytopathic Effect Assay on MDCK-2 Cells and Measurement of the Photocatalytic Activity

The cytopathic effect was assessed by two independent 2 × 10^4^ cells per well that were seeded in two transparent 96 well plates with complete medium and incubated overnight. The day after, the supernatant was discarded, and cells were treated with serial dilution of photocatalyst ranging from 10 ng/mL to 0.01 mg/mL. Cells were covered with aluminum foil or irradiated with white light source for two hours. The cells were incubated at 37 °C with 5% CO_2_. After 72 h, 20 μL of 3-(4,5-dimethylthiazol-2-yl)-2,5-diphenyl-2H-tetrazolium bromide (Sigma-Aldrich) dissolved in PBS at 7.5 mg/mL was added to each well and incubated at 37 °C with 5% CO_2_ for 1 h. Then, the supernatant was removed, and cells were lysed with 100 μL/well of 10% 2-Propanol, 0.004% Triton-X-100 (Sigma-Aldrich), 0.0004% HCl, then the absorbance was read at 570 nm with a plate reader Victor Nivo5 PerkinElmer (Waltham, MA, USA).

Viral titration. Plaque assay. A total of 4 × 10^5^ MDCK-2 cells per well were seeded in 12 well plates in a complete medium the day after the supernatant was discarded, and cells were treated with serial dilution of virus in DMEM solo and incubated for 1 h. Later the virus was removed, and cells were covered with 2 mL/well of immobilizing media (DMEM + 1% KAN, 0.1% trypsin, and 1% low melting agar) and incubated for 72 h. After the immobilizing overlay was removed, the cells stained with 0.8% crystal violet in 50% Ethanol and the plaques were counted by visual inspection.

Viral Infectivity Reduction assay of PhCN/TiO_2_ on MDCK-2 cell line. Additionally, 600 PFU of Influenza Virus (H1N1) strain A/Puerto Rico/8/34 (H_1_N_1_) were incubated with 1.25 mg/mL of PhCN/TiO_2_ suspension on DMEM solo, and a control solution without PhCN/TiO_2_ suspension was prepared. Solution was split into two parts, where one part was irradiated and the other incubated in the dark for two hours. Viral titer variation was determined by titration via plaque assay.

## 3. Results and Discussion

**Figure 1:**
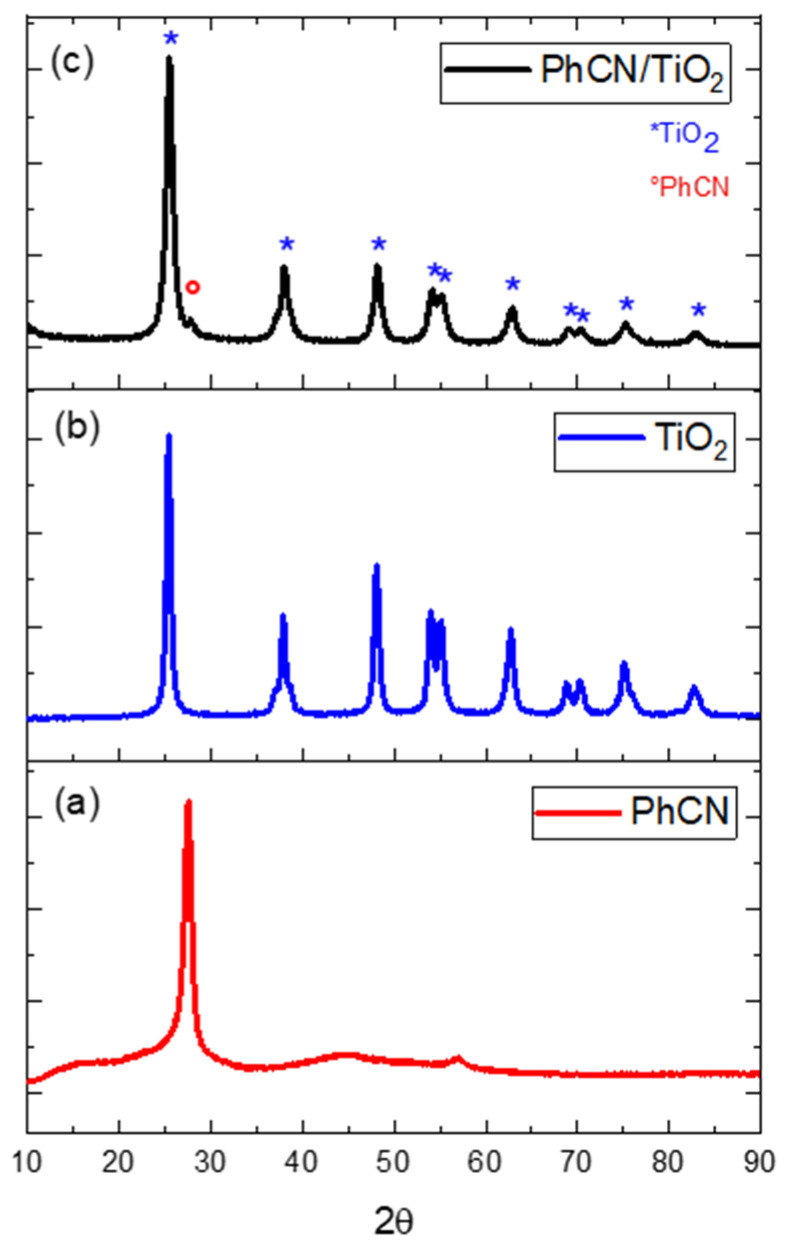
X-ray diffraction patterns of PhCN (**a**), TiO_2_ (**b**), and PhCN/TiO_2_ (**c**).

Figure 1 reports the X-ray diffraction patterns of PhCN/TiO_2_ (c) and, for comparison, the diffraction pattern of pure PhCN (a) and TiO_2_ (b).

According to [27] the XRD pattern of PhCN (phenyl carbon nitride) obtained at 400 °C shows two broad bands located at approximately 15° and 27.6° 2θ. The peak located at the higher angle (27.6°) is associated with the (001) reflection, which is indicative of the distance between the layers in the crystal structure. This peak suggests that PhCN adopts a layered structure, in which the heptazinic chains are stacked parallel to each other and separated by a distance that corresponds to the (001) plane spacing. The peak at the lower angle (15°) is associated with the (210) reflection, which is indicative of the distance between adjacent heptazinic chains within each layer. This peak suggests that the heptazinic chains are oriented perpendicular to the layers and are packed in a regular manner within each layer (Figure 1a).

In the case of anatase TiO_2_, the most intense peaks are typically located at 25.3°, 37.8°, 47.9°, 54.1°, 62.7°, 68.8°, and 75.1° 2θ (where 2θ is the diffraction angle). These peaks are commonly labeled as (101), (004), (200), (105), (211), (204), and (215), respectively (Figure 1b). The crystal phase of TiO_2_ is fundamental in defining its photocatalytic properties and anatase possesses the highest efficiency compared to Rutile and brookite [21]. This is due to its unique crystal structure, which allows for efficient charge separation and transfer, leading to the generation of reactive oxygen species that can degrade organic pollutants [28].

The diffraction pattern of the hybrid material provides information about the crystalline structure of the individual components and their interaction in the hybrid system. In particular, the presence of diffraction peaks corresponding to both the organic and inorganic components indicates that the two materials are well-mixed and have formed a single phase (Figure 1c). The XRD pattern of the PhCN/TiO_2_ hybrid system reports the diffraction peak of anatase, and no secondary phases are observed; in addition, the peak principal peak of PhCN is detectable in the pattern. The interaction between the two structures assumes a fundamental importance in the photocatalytic process facilitating the charge transfer and separation. Additionally, the presence of a single inorganic phase ensures that the hybrid material has a homogeneous composition and distribution of the components, which strongly minimizes the presence of trapping sites.

**Figure 2:**
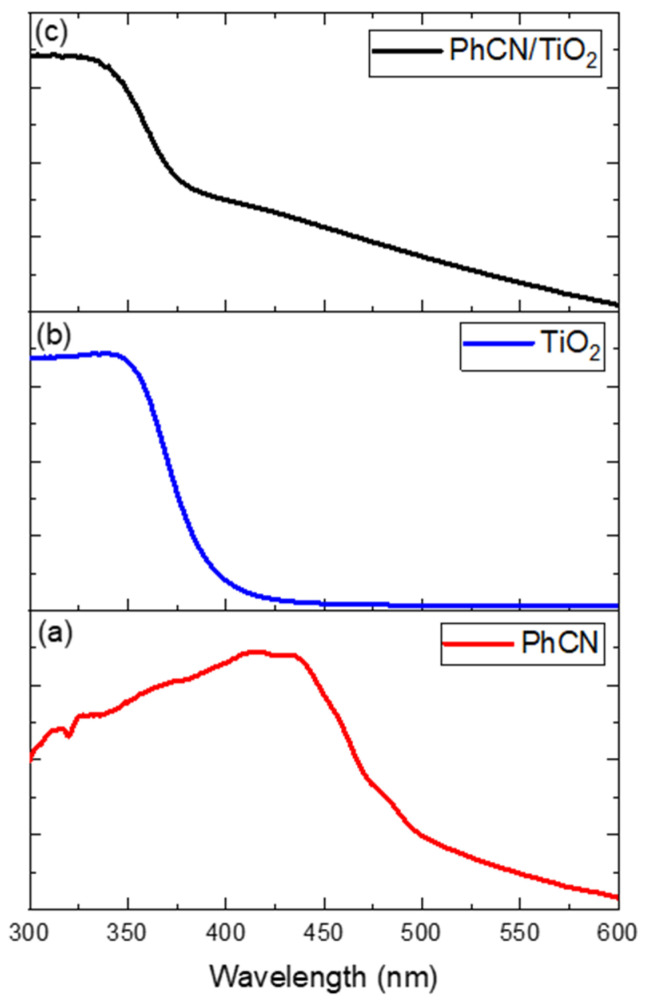
Absorption spectra of PhCN (**a**), TiO_2_ (**b**), and PhCN/TiO_2_ (**c**).

The UV–Vis absorption spectra of the two components used in the hybrid system (TiO_2_ and PhCN) are shown in Figure 2a,b. TiO_2_ in its anatase form is a well-known semiconductor material that has attracted significant attention for its photocatalytic properties. It has a relatively wide band gap of 3.2 eV, and it mainly absorbs in the UV region (<410 nm). On the other hand, PhCN is an organic compound that contains phenyl groups, which give it a broad absorption band starting at around 600 nm and extending, consequently, the absorption of the hybrid system into the visible region, too (Figure 2c).

**Figure 3:**
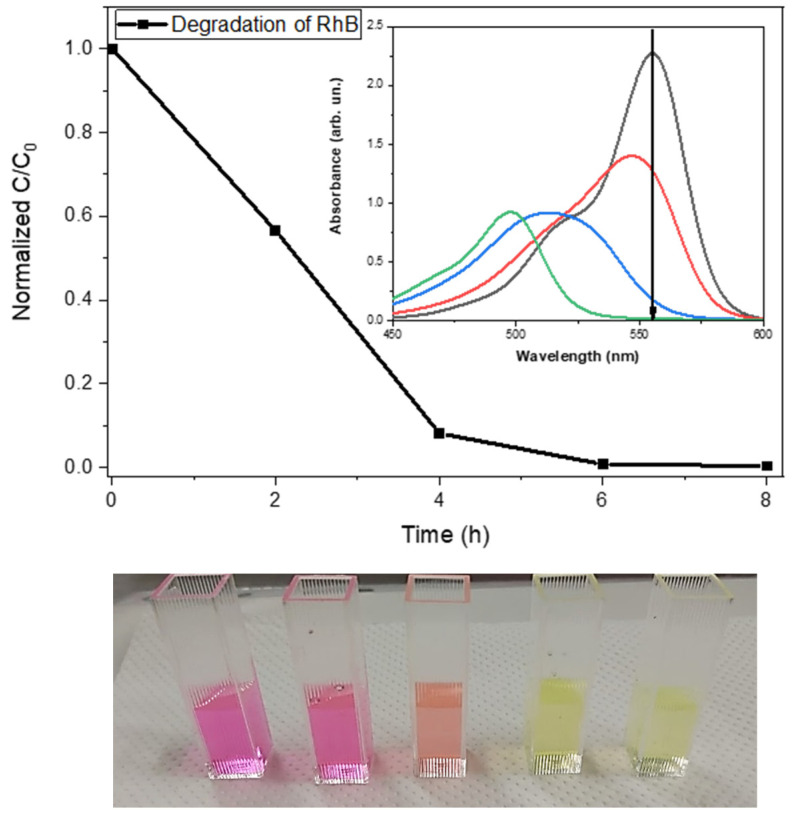
Degradation of Rhodamine B under visible light irradiation. The graph represents the decrease in concentration of the Rhodamine during the time. The caption shows the absorption spectra collected at different time intervals and the arrow indicates how the degradation of the Rhodamine occurs showing both a decrease of the intensity and a shift.

Visible light constitutes a significant portion of the solar spectrum and is readily available for energy conversion. Indeed, the photocatalytic efficiency of the PhCN/TiO_2_ hybrid material has been demonstrated in the degradation of a solution of Rhodamine B dye (10 mg/L) under visible light illumination from a white LED source (11 W, 0.2 mW/cm^2^). The complete degradation of the dye was achieved in just 6 h, highlighting the efficacy of the material as a visible light photocatalyst. (Figure 3). The photoinduced electron transfer (PET) mechanism observed in the PhCN/TiO_2_ hybrid material is a crucial process that enables the material to exhibit photocatalytic activity. The mechanism involves the absorption of visible light by the organic component, which then promotes an electron from the highest occupied molecular orbital (HOMO) ground state to the lowest unoccupied molecular orbital (LUMO) excited state. This process creates a photoexcited state in the organic component, which can then transfer an electron to the conduction band of the TiO_2_ semiconductor.

The transfer of the electron to the TiO_2_ semiconductor is a critical step in the PET mechanism, as it creates a charge separation between the organic and inorganic components. This charge separation allows the material to act as a photocatalyst, as the photoexcited electrons in the TiO_2_ conduction band can react with adsorbed molecules on the surface of the material, leading to various chemical transformations. The holes left in the valence band of the TiO_2_ semiconductor can also participate in redox reactions and react with other molecules in the surrounding environment.

### 3.1. Photocatalytic Inactivation of the Virus

#### 3.1.1. Cytotoxicity Test

The photocatalytic degradation of viruses was tested using a heterojunction system consisting of Phenyl carbon nitride/TiO_2_ as the photocatalyst, a white-LED lamp (11 W, 0.2 mW/cm^2^) to activate the process, MDCK cells, and H1N1 flu virus.

The initial studies focused on understanding the cytotoxic activity of the photocatalyst as a function of its concentration. The MTT method was used to assess the test [29].

**Figure 4:**
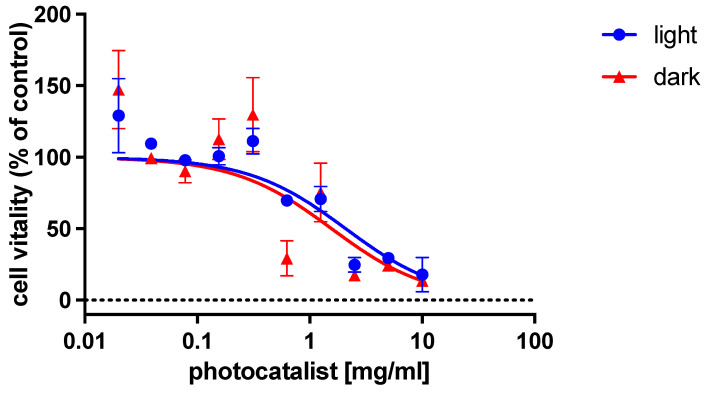
Direct cytotoxic effect of PhCN/TiO_2_ on MDCK-2 cell line.

As shown in Figure 4, the test was conducted under both light and dark conditions to study the efficiency of the photocatalyst and to exclude the interaction of the photocatalyst and light separately with the cells.

Different concentrations of photocatalysts ranging from 10 mg/mL to 0.020 mg/mL were tested with a 0.5 dilution factor. The test results indicated that the best concentration range for non-cytotoxic photocatalyst activity was between 1.25 and 0.020 mg/mL (Figure 4). Afterwards, the major concentration allowed (1.25 mg/mL) was utilized to perform the plaque assays.

#### 3.1.2. Virus Inactivation

**Figure 5:**
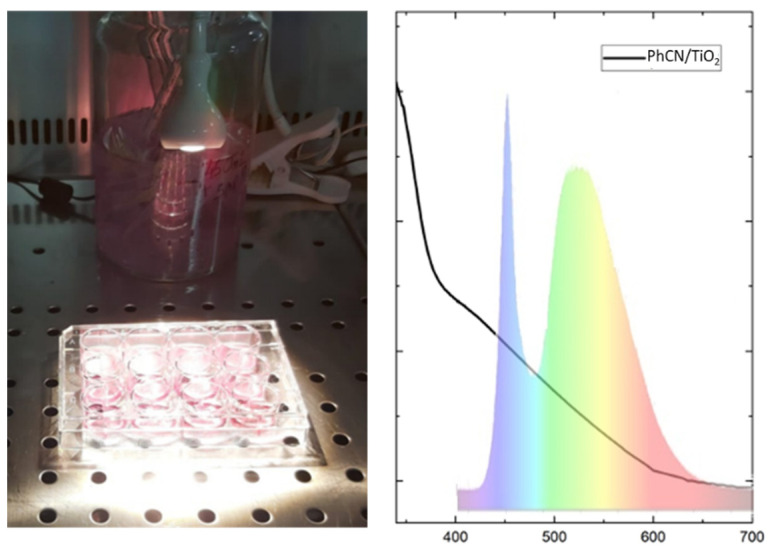
Photocatalytic virus disinfection experiment with an illustration of the absorption spectrum of the material compared to the emission spectrum of the LED.

To test the effectiveness of the photocatalyst in degrading the virus, 600 PFU of Influenza Virus (H1N1) were mixed with 1.25 mg of photocatalyst. The solution was split in two, and half of the solution was irradiated for 2 h using a 1white-LED lamp (optical flux of 0.2 mW/cm^2^, generated by 11 W electrical power, Figure 5), and the other half was covered with aluminum foil for 2 h. Figure 5 shows the emission spectrum of the lamp and its overlapping with the absorption response of the hybrid system. It is worth pointing out that there is not any UV component involved in the reaction, further indicating that the inhibition of the virus is not light-induced and the possibility of utilizing the interior light to activate the photocatalytic process.

After the irradiation, plaque assays were performed by inoculating different dilutions of the virus solution (1:1, 1:10, 1:100) onto cell monolayers, both with and without the photocatalyst. The monolayers were incubated at 37 °C in a 5% CO_2_ atmosphere for 1 h to allow the virus to attach to the cells.

After the incubation time, the suspension was removed, and the monolayers were covered with an immobilizing solution containing agar, which caused the formation of a gel. The monolayers were then incubated again for 72 h, during which time each infectious particle produced a circular zone of infected cells called a plaque. To identify the plaques, the cell monolayers were stained with 500 mL of crystal violet and the plaques were counted after washing out and drying.

We further tested two control solutions of (i) virus without photocatalyst and (ii) photocatalyst without virus. The solutions were irradiated or incubated in the dark, respectively.

The results of this test were presented in pfu/mL, which represents the number of infectious particles in the sample, assuming that each plaque was caused by a single infectious virus particle. By multiplying the number of observed plaques by the dilution factor of the virus solution, the virus titer could be calculated.

Overall, the plaque assay is a widely used method to quantify the number of infectious particles in a viral sample and is based on the ability of viruses to infect and lyse host cells. By using this assay in combination with the photocatalyst, the effectiveness of the photocatalyst in degrading the virus could be assessed.

**Figure 6:**
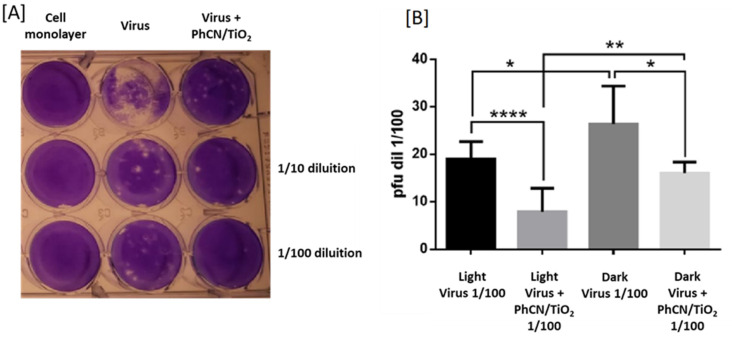
(**A**) Representative result of a plaque assay titrating the samples obtained after irradiation procedure. Column1 MDCK-2 control wells with PhCN/TiO_2_, Column 2 Virus control dilution without photocatalyst, Column 3 Virus dilution treated with photocatalyst. (**B**) Result of a plaque assay titrating the samples obtained after irradiation procedure. Plaque-forming units were quantified by visual inspection. Statistical significance was determined with an unpaired-*t* test using Prism 9.01.

Figure 6A displays the results of a plaque assay conducted to compare the efficacy of different conditions. The control condition involved the addition of only the photocatalyst solution (at a concentration of 1.25 mg/mL) to the cell monolayer, which resulted in no observable plaques, indicating that the photocatalyst alone is not toxic to the cells.

The middle column represents the number of plaques formed when only the virus was added to the cell monolayer, which serves as a control to compare the number of plaques formed in the presence of the photocatalyst. The right column shows the number of plaques formed when the virus was added to the cell monolayer in the presence of the photocatalyst.

Since the number of plaques observed in the presence of the photocatalyst was lower than the number of plaques observed without the photocatalyst, it is possible to argue that the photocatalyst has virucidal activity. Furthermore, it reduces the infectivity of the virus particles by damaging the virus’s genetic material or its outer envelope, rendering them unable to infect cells.

To ensure that the observed effect was not due to the amount of photocatalyst added or to any other experimental artifact, the experiment was performed at three different concentrations, confirming that the reduction in plaque formation is indeed due to the virucidal activity of the photocatalyst.

It is interesting to note that the experiment was conducted using MDCK cells and H1N1 virus, which are commonly used in influenza virus research. By testing the effect of photocatalyst on the virus in both light and dark conditions, we were able to determine the role of light in the virucidal process (Figure 6B).

The use of a *t*-test is a common statistical method used to compare two sets of data and determine if the observed differences are statistically significant. In this case, the *t*-test (Table 1) was used to compare the number of plaques observed under different conditions, and the results showed a significant difference between all run tests. The *p*-value is a statistical measure that indicates the level of significance of the results obtained from a *t*-test. In general, a *p*-value of less than 0.05 is considered statistically significant, which means that the probability of obtaining the observed difference by chance alone is less than 5%. Therefore, if the *p*-value is less than 0.05, it indicates that there is a statistically significant difference between the groups being compared.

Results indicate that the presence of the photocatalyst and/or light significantly affects the number of plaques formed, suggesting that the photocatalyst is an effective virucidal agent against the H1N1 virus.

In the context of the present study, the significant *p*-values obtained from the *t*-tests indicate that there are significant differences between the various experimental conditions tested. For instance, the comparison between the virus in light versus the virus in the dark and the virus plus photocatalyst in light versus the virus plus photocatalyst in the dark yielded statistically significant *p*-values. This suggests that light has a role in the virucidal effect of the photocatalyst, with a reduction of over 50% of viral titer present in the solution after only 2 h of treatment.

Moreover, the *t*-test comparing dark conditions with virus plus photocatalyst versus virus alone and light conditions with virus plus photocatalyst versus virus alone yielded statistically significant *p*-values. This confirms that the presence of the photocatalyst has a significant effect on reducing the number of plaques formed by the H1N1 virus in MDCK cells.

Overall, the significant *p*-values obtained from the *t*-tests provide strong evidence that the photocatalyst is an effective virucidal agent against the H1N1 virus in MDCK cells, and its effect is modulated by light. Overall, this experiment provides valuable information on the potential use of photocatalysts as virucidal agents and highlights the importance of studying the role of light in such processes.

## 4. Conclusions

In summary, the findings of this study suggest that the polymer-based hybrid photocatalyst (phenyl modified Carbon Nitride/TiO_2_) is a highly effective virucidal agent against the H1N1 virus. The results of the plaque assay demonstrate that the presence of the photocatalyst significantly reduces the number of virus-induced plaques formed in the cell monolayer, indicating that the photocatalyst can inactivate the virus.

The measurements, conducted under low visible light flux, indicate that the photocatalyst is a reliable and effective virucidal agent against the H1N1 virus, suggesting its use in indoor environments such as hospitals or public places where direct sunlight exposure may be limited.

Further studies are required to investigate the mechanism of action of the photocatalyst and its potential use as an antiviral agent against other viral strains. Nonetheless, the promising results of this study suggest that the photocatalyst could be a valuable addition to the arsenal of antiviral agents available to combat viral infections.

## Figures and Tables

**Table 1:** Results of the *t*-test *p*-values for the tested conditions.

	No Photocatalyst	Photocatalyst	Photocatalyst (Dark)	Photocatalyst (Light)
	Dark v 1:100 vs. Light v 1:100	Dark v + photocatalyst 1:100 vs. Light v + photocatalyst 1:100	Dark v + photocatalyst 1:100 vs. Dark v + 1:100	Light v + photocatalyst 1:100 vs. Light v + 1:100
*p* value	0.032	0.0051	0.0231	<0.0001
*p* value summary	*	**	*	****
Significantly different? (*p* < 0.05)	Yes	Yes	Yes	Yes

## Data Availability

The datasets generated during and/or analysed during the current study are available from the corresponding autor on reasonable request.
